# *Acinetobacter baumannii*: an evolving and cunning opponent

**DOI:** 10.3389/fmicb.2024.1332108

**Published:** 2024-01-22

**Authors:** Jingchao Shi, Jianghao Cheng, Shourong Liu, Yufeng Zhu, Mingli Zhu

**Affiliations:** ^1^Open Laboratory Medicine, Hangzhou Xixi Hospital Affiliated to Zhejiang Chinese Medical University, Hangzhou, China; ^2^Department of Clinical Laboratory, Affiliated Jinhua Hospital, Zhejiang University School of Medicine, Jinhua, China; ^3^Graduate School, Zhejiang Chinese Medical University, Hangzhou, China; ^4^Department of Infectious Disease, Hangzhou Xixi Hospital Affiliated to Zhejiang Chinese Medical University, Hangzhou, China

**Keywords:** *Acinetobacter baumannii*, resistance mechanisms, tigecycline, polymyxin, antimicrobial resistance

## Abstract

*Acinetobacter baumannii* is one of the most common multidrug-resistant pathogens causing nosocomial infections. The prevalence of multidrug-resistant *A. baumannii* infections is increasing because of several factors, including unregulated antibiotic use. *A. baumannii* drug resistance rate is high; in particular, its resistance rates for tigecycline and polymyxin—the drugs of last resort for extensively drug-resistant *A. baumannii*—has been increasing annually. Patients with a severe infection of extensively antibiotic-resistant *A. baumannii* demonstrate a high mortality rate along with a poor prognosis, which makes treating them challenging. Through carbapenem enzyme production and other relevant mechanisms, *A. baumannii* has rapidly acquired a strong resistance to carbapenem antibiotics—once considered a class of strong antibacterials for *A. baumannii* infection treatment. Therefore, understanding the resistance mechanism of *A. baumannii* is particularly crucial. This review summarizes mechanisms underlying common antimicrobial resistance in *A. baumannii*, particularly those underlying tigecycline and polymyxin resistance. This review will serve as a reference for reasonable antibiotic use at clinics, as well as new antibiotic development.

## 1 Introduction

*Acinetobacter baumannii*, an aerobic, nonfermenting, gram-negative, rod-shaped bacterium, is commonly found in water, soil, air, and other natural environments; it can also colonize the skin, conjunctiva, oral cavity, respiratory tract, gastrointestinal tract, and genitourinary tract of healthy individuals (Dexter et al., 2015; Carvalheira et al., 2021). *A. baumannii* demonstrates strong adhesion and ability to colonize various surfaces; moreover, it is resistant to not only dry and moist conditions but also general disinfectants and ultraviolet radiation. These characteristics render *A. baumannii* a major pathogen, frequently causing opportunistic nosocomial infections, including wound infections, central nervous system infections, abdomen infections, urinary tract infections, and bacteremia, in immunocompromised patients (Munoz-Price and Weinstein, 2008). According to the CHINET’s bacterial drug resistance surveillance data, *A. baumannii* was one of the top five predominantly distributed surveilled strains, as well as the second leading nonfermenting, gram-negative bacterium in China. Moreover, 71.5 and 72.3% of the *A. baumannii* strains were resistant to imipenem and meropenem, respectively, and ≥50% of the *A. baumannii* strains were resistant to other antimicrobials. In other words, *A. baumannii* has strong drug resistance and the ability to transmit clonally (Hu et al., 2022). Uncontrolled antibiotic use has led to global epidemics caused by multidrug-resistant, extensively drug-resistant, and pandrug-resistant *A. baumannii* (Bassetti et al., 2018; Assimakopoulos et al., 2023). Because it can elude the effects of antimicrobials, the World Health Organization has designated *A. baumannii* as one of the most dangerous ESKAPE pathogens; other ESKAPE pathogens include *Enterococcus faecalis*, *Staphylococcus aureus*, *Klebsiella pneumoniae*, *Pseudomonas aeruginosa*, and *Enterobacter* spp. (Denissen et al., 2022). Plasmid splicing, integrons, transposons, and other mechanisms are involved in the clonal transfer of drug resistance genes between different strains (Chen et al., 2017; Ambrose et al., 2023). The increase in antimicrobial resistance has resulted in a global public health emergency (Amann et al., 2019). In particular, the increase in *A. baumannii* antibiotic resistance has led to major obstacles in clinical treatment. Moreover, bacterial drug resistance has become a focus of clinical medicine and public health security. *A. baumannii* demonstrates considerable resistance to many antibiotics, which makes combination therapy the mainstay of current clinical treatment. However, no combination therapy has been noted to significantly lower mortality or enhance clinical response in patients with *A. baumannii* infections. To develop novel antibiotics and manage the spread of illness effectively, thoroughly comprehending the mechanisms underlying *A. baumannii* antibiotic resistance is crucial.

## 2 *Acinetobacter baumannii* drug resistance mechanisms

**Figure 1:**
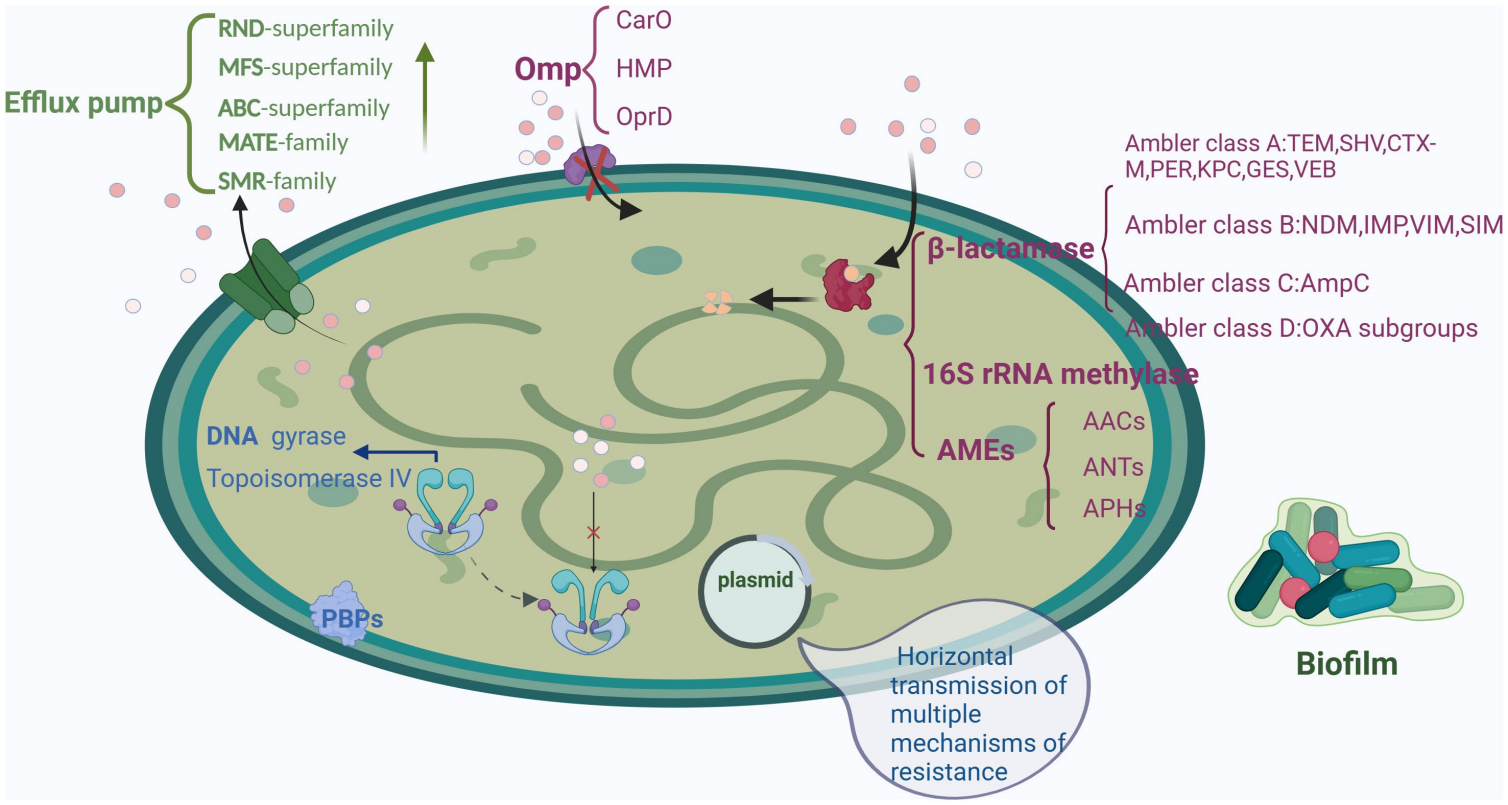
Mechanisms underlying drug resistance in *A. baumannii*: β-lactamase production, AMEs, and 16S rRNA methylase, efflux pump overexpression, antibiotic target location alterations, Omp permeability alterations, and biofilm formation. Created with BioRender.com.

Compared with other bacteria, *A. baumannii* has a highly sophisticated resistance mechanism. For instance, it includes various antimicrobial-inactivating enzymes (particularly β-lactamases) and efflux pump overexpression, as well as alterations in antibiotic target location and outer membrane protein permeability (Table 1, Figure 1).

**Table 1:** Mechanism of drug resistance in *Acinetobacter baumannii*.

**Resistant mechanism**	**Class or subgroup**	**Gene**	**References**
β-Lactamases	Ambler class A	TEM, SHV, PER, VEB, KPC	Černiauskienė et al. (2023)
	Ambler class B	IMP, VIM, SIM, NDM	Chen et al. (2017), López et al. (2019), Ayibieke et al. (2018)
	Ambler class C	AmpC	Liu and Liu (2015)
	Ambler class D	OXA23 family OXA24 family OXA51 family OXA58 family OXA143 family OXA235 family	Lin and Lan (2014), June et al. (2016), Huang et al. (2015), Donald et al. (2000), Avci et al. (2023), Zhao et al. (2019), Oliveira et al. (2019), Liao et al. (2015)
Aminoglycoside modifying enzymes	Aminoglycoside acetyltransferases (AACs)	AAC(3)-I AAC(3)-II AAC(6′)-I ANT(2″)-I ANT(2″)-II APH(3′)-I APH(3″)-II	Lin and Lan (2014), Lin et al. (2015), Sheikhalizadeh et al. (2017)
	Aminoglycoside nucleotidyltranferases (ANTs)		
	Aminoglycoside phosphotransferases (APHs)		
	16S rRNA methylase	armA	Gholami et al. (2017)
Changing the target locations	DNA gyrase and topoisomerase	gyrA, parC	Gu et al. (2015)
	Penicillin-binding protein (PBPs)	PBP2, PBP1	Cochrane and Lohans (2020), Toth et al. (2022)
Formation of biofilms	Biofilms	BfmRS, abaI, epsA, pglC, ompA	Geisinger et al. (2018), Yang et al. (2019), Romero et al. (2008)
Overexpression of the external discharge pumping system	RND superfamily	AdeABC AdeIJK AdeDE AdeFGH	Yoon et al. (2013), Modarresi et al. (2015), Chang et al. (2016), Lin et al. (2014), Meyer et al. (2022), Darby et al. (2023), AlQumaizi et al. (2022), Hou et al. (2012)
	MFS superfamily	TetA, TetB	Lomovskaya et al. (2018), Sumyk et al. (2021)
		CraA	Foong et al. (2019)
		AmvA	Rostami et al. (2021)
	MATE family	AbeM	Srinivasan et al. (2015)
	SMR family	AbeS	Lytvynenko et al. (2016)
	ABC superfamily	MsbA	Hammerstrom et al. (2015)
Changes in outer membrane pore proteins	Omp	CarO, HMP, OprD	Wu et al. (2016), Smani et al. (2014), Fonseca et al. (2013)
**Mechanisms of resistance to tigecycline**			
Efflux pump	RND superfamily	AdeABC AdeIJK AdeFGH	Jo and Ko (2021), Salehi et al. (2021), Hornsey et al. (2010), Lin et al. (2014), Gerson et al. (2018), Deng et al. (2014), Li et al. (2017)
	ABC superfamily	MsbA	Chen et al. (2014)
		MacAB-TolC	Lin et al. (2017b)
	MFS superfamily	tetA, tet(Y)	Wang et al. (2021) Liu et al. (2023)
	AcrAB-TolC	hns	
Modified enzyme mediated	Tet(X)	Tet(X), Tet(X3), Tet(X5), Tet (X6)	Cui et al. (2021), He et al. (2019), Chen et al. (2021)
Change in outer membrane permeability		plsC abrP gnaA	Li et al. (2015), Li et al. (2016), Xu et al. (2019)
Changes in drug targets		rpsJ trm rrf	Hammerstrom et al. (2015), Beabout et al. (2015), Chen et al. (2014), Ghalavand et al. (2022), Zheng et al. (2023), Hua et al. (2021)
DNA damage-induced reactions		recA, recBCD	Ajiboye et al. (2018)
**Mechanisms of resistance to colistin**			
Modification of LPS structures	PEtN	PmrA, PmeB, PmrC EptA	Olaitan et al. (2014), Potron et al. (2019), Sun et al. (2020), Kabic et al. (2023), Nurtop et al. (2019), Gerson et al. (2020) Trebosc et al. (2019), Ye et al. (2023)
		mcr-4.3, mcr-1, mcr-2, mcr-3	Martins-Sorenson et al. (2020), Snyman et al. (2021), Ma et al. (2019), Al-Kadmy et al. (2020)
	Galactosamine	NaxD	Chin et al. (2015)
Loss of LPS		lpxA, lpxC, lpxD	Moffatt et al. (2011), Nurtop et al. (2019), Jovcic et al. (2021), Thi Khanh Nhu et al. (2016), Carretero-Ledesma et al. (2018), Wand et al. (2015)
Overexpression of efflux pumps	EmrAB effector pump system	emrB	Lin et al. (2017a), Ni et al. (2016)
Mutations in outer membrane constituent proteins	Non-LPX proteins lipoprotein	lpsB LptD VacJ, PldA	Hood et al. (2013), Dafopoulou et al. (2015), Lean et al. (2014), Yang W. et al. (2023); Yang Y. et al. (2023) Bojkovic et al. (2015) Thi Khanh Nhu et al. (2016)

### 2.1 Mechanisms underlying β-lactamase production

β-Lactamases destroy the β-lactam rings of penicillins, cephalosporins, monocyclics, and carbapenems, rendering them inactive and without their antibacterial properties. Most *A. baumannii* strains produce β-lactamases as their primary method of defense against β-lactam antibiotics. β-Lactamases can be divided into classes A, B, C, and D according to Ambler’s molecular structure categorization (i.e., sequence characterization of β-lactamase’s amino-terminal end) (Lee et al., 2017).

Serine proteases such as penicillinase, cephalosporinase, carbenicillin hydrolase, and extended-spectrum β-lactamases (ESBLs) are class A β-lactamases. Most ESBLs are class A enzymes, whereas a few of them are class D enzymes. The several types of class A ESBLs include TEM, SHV, CTX-M, and others (e.g., PER, KPC, VEB, GES, BES, and SFO). Černiauskienė et al. (2023) detected TEM, SHV, PER, VEB, KPC, and other ESBLs in *A. baumannii*. TEM-type ESBLs, detected most frequently in *A. baumannii*, are the main mechanisms underlying β-lactam antibiotic resistance in *A. baumannii*. *A. baumannii* may hydrolyze carbapenems, first discovered in the United States and now found in many other countries (Robledo et al., 2010). Furthermore, GES-22 ESBL-producing *A. baumannii* has developed resistance to even typical ESBL inhibitors, such as sulbactam, clavulanate, and tazobactam; in particular, it exhibits high potassium clavulanate and sulbactam hydrolysis efficiency (Saral et al., 2016). Chromosomes and plasmids are the primary carriers of class A β-lactamase genes; moreover, these genes may be acquired through conjugation, transformation, or transduction.

Class B β-lactamases are also called metallo-β-lactamases (MBLs) because they have catalytically active centers that require the involvement of metal ions such as Zn^2+^. MBLs extensively hydrolyze most anti-β-lactamase medications, which also decreases their bactericidal activity. Imipenem has a particularly large hydrolysis capacity. Monocyclic antibiotics, including aztreonam (ATM), are an exception to this. MBLs are also resistant to conventional enzyme inhibitors such as sulbactam, tazobactam, and clavulanate; they can be inhibited by metal chelating compounds, which bind to metal ions and ethylene diamine tetraacetic acid (López et al., 2019). *A. baumannii* MBLs can be mainly divided into IMP, VIM, SIM, and NDM types. New Delhi MBL (NDM-1) is a highly effective enzyme, recently discovered to have a large capacity to hydrolyze carbapenem medicines, and bacteria bearing the NDM-1 gene exhibit pandrug resistance. Therefore, NDM-1-carrying bacteria are also called superbugs. The NDM-1 gene is mainly located on the bacterial plasmid, with a small portion located on their chromosome (Ayibieke et al., 2018). Chen et al. (2017) demonstrated that the NDM-1 gene can be transmitted from one strain to another through plasmids and that plasmids play a key role in the outbreak of pandrug-resistant *A. baumannii*. Therefore, particular attention must be toward the spread of NDM-1-carrying *A. baumannii*.

The occurrence of newer MBLs has been reported in recent years. Currently, cefiderocol or the combination of CZA and ATM (CZA/ATM) is the preferred first-line treatment for infections of bacteria with MBL-mediated resistance. However, bacteria have developed resistance to cefiderocol, CZA/ATM, or both; the currently available data indicate that the effectiveness of cefiderocol and CZA/ATM against MBL-producing bacteria is inadequate, and treatment is linked to a high (albeit decreasing) mortality rate. Consequently, the therapeutic requirement for MBL inhibitors, particularly related to primary clinically significant MBLs, remains unfulfilled. Nevertheless, researchers are currently focused on creating clinically effective MBL inhibitors, such as those with metal-binding pharmacophores, which serve as coordination or a bridge between Zn_1_ and Zn_2_ (Yang W. et al., 2023; Yang Y. et al., 2023).

Class C β-lactamases, also known as AmpC enzymes or cephalosporinases, target cephalosporins and are not inhibited by clavulanate. AmpC enzymes are the primary reason that *A. baumannii* develop resistance to third-generation cephalosporins; they are encoded by the *A. baumannii* chromosome at a high carrier rate. AmpC enzymes are expressed more abundantly when the insertion sequence ISAba1 is present upstream of their genes. AmpC enzymes are divided into chromotype (ADC) and plasmid-type (DHA). *Acinetobacter*’s chromosomal *ampC* may have descended from a common β-lactamase gene progenitor, and it shares a closer relationship with *Acinetobacter* than other bacteria (Liu and Liu, 2015). Infections of *A. baumannii* that produces AmpC enzymes are currently treated using carbapenems and fourth-generation cephalosporins; nevertheless, attention should be made to the prevalence of AmpC enzyme–producing *A. baumannii*.

Class D β-lactamases are also called oxacillin (OXA) enzymes because of their ability to hydrolyze semisynthetic penicillins, such as oxacillin. They also have hydrolytic activity against cephalosporins and carbapenems. These β-lactamases feature has carboxylated lysine at its center, which leads to its distinct catalytic mechanism. The presence of MBLs and OXA enzymes is a main mechanism underlying carbapenem resistance in *A. baumannii* (Lin and Lan, 2014).

OXA enzymes prevalent in *A. baumannii* mainly include OXA23 and OXA24 family enzymes, as well as OXA51, OXA58, OXA143, and OXA235. Of them, OXA51 occurs naturally in *A. baumannii* but with weak carbapenem-hydrolyzing ability (June et al., 2016). In a UK study, the OXA51 gene was found in all *A. baumannii* isolates, and the presence of an ISAba1 insertion sequence in the OXA51 gene enhanced carbapenem hydrolysis (Huang et al., 2015). OXA23, the first class D β-lactamase that can hydrolyze carbapenems through plasmid delivery, was originally known as *Acinetobacter* resistant to imipenem (ARI) 1 (Paton et al., 1993; Scaife et al., 1995; Donald et al., 2000). In reality, OXA23 can be put into chromosomes or plasmids by using various genetic elements as vectors, including transposons and insertion sequences (Avci et al., 2023). The rate of drug-resistant organisms increases with the number of mobile elements found simultaneously in OXA23-positive *A. baumannii*, which is enhanced by the presence of ISAba1 (Oliveira et al., 2019; Zhao et al., 2019). The presence of OXA23 is the main reason that *A. baumannii* develops carbapenem resistance. *A. baumannii* may transfer the OXA24 gene to other strains by secreting vesicles from its outer membrane (Rumbo et al., 2011). In addition, *A. baumannii* outer membrane vesicles facilitate OXA58 gene transfer and OXA58 release. The presence of carbapenems enhances OXA58 vesicle release, and the vesicles can protect carbapenem-sensitive *A. baumannii* (Liao et al., 2014, 2015).

### 2.2 Aminoglycoside-modifying enzymes and 16S rRNA methylase

The major mechanism underlying *A. baumannii*’s resistance to aminoglycosides is aminoglycoside-modifying enzyme (AME) and 16 s rRNA methylase production. AMEs can be divided into three categories: aminoglycoside acetyltransferases (AACs), aminoglycoside nucleotidyltranferases (ANTs), and aminoglycoside phosphotransferases (APHs), encoded by *aac*, *ant*, and *aph*, respectively. These enzymes are typically found in transposons, integrons, and plasmids and are responsible for the spread of drug-resistant genes (Lin and Lan, 2014). APHs phosphorylate the free hydroxyl groups, whereas both AACs and ANTs acetylate them. AMEs detected in *A. baumannii* include AAC(3)-I, AAC(3)-II, AAC(6′)-I, ANT(2″)-I, ANT(2″)-II, APH(3′)-I, and APH(3″)-II. Drug resistance results from AMEs’ ability to modify particular aminoglycoside groups, change covalent bonding, block medications from attaching to the target ribosome, and prevent bacterial protein production.

Many multidrug-resistant *A. baumannii* isolates have demonstrated various combinations of AMEs. Lin et al. (2015) reported that five aminoglycoside resistance genes coexisted in 37.5% of aminoglycoside-resistant *A. baumannii*. Drugs and resistance genes are correlated in varied ways: AAC(3′)-Ia is associated with tobramycin and amikacin resistance; AAC(3′)-IIa is associated with kanamycin resistance; ANT(2′)-Ia is associated with tobramycin, gentamicin, kanamycin, and amikacin resistance; and APH(3′)-Via is associated with amikacin and kanamycin resistance (Sheikhalizadeh et al., 2017).

Another major aminoglycoside resistance mechanism in *A. baumannii* is 16S rRNA methylation; it reduces the affinity of medications for aminoglycosides (Gholami et al., 2017). Examples of common 16S rRNA methylase genes include *armA*, *rmtA*, *rmtB*, *rmC*, *rmtD*, *rmtE*, *rmtF*, *rmtG*, *rmtH*, and *npmA*. *A. baumannii* possesses many armA genes, possibly contributing to the organism’s resistance to aminoglycosides.

### 2.3 DNA gyrase and topoisomerase gene mutations and plasmid quinolone resistance genes

Quinolones are synthetic antimicrobial drugs with the basic structure of 4-quinolones. They demonstrate antimicrobial effects by inhibiting bacterial DNA replication, specifically targeting topoisomerase IV and DNA gyrase. The tetramer DNA gyrase is composed of two A subunits (GraA) and two B subunits (GraB), encoded by *gyrA* and *gyrB*, respectively. DNA breaks and rejoins when a DNA gyrase subunit is present; in particular, ATP hydrolysis is catalyzed by a B subunit. Topoisomerase IV has two C subunits, encoded by *parC*, and two E subunits, encoded by *parE*. In *A. baumannii*, quinolone resistance is primarily caused by mutations in *gyrA* and *parC*. Quinolones cannot bind to DNA gyrase and topoisomerase IV with structural changes in complexes, preventing DNA replication and leading to drug resistance (Gu et al., 2015). Mutations in *gyrA* single genes lead to moderate resistance, whereas those in *gyrA* and *parC* result in high resistance.

A novel mechanism underlying drug resistance, revealed in recent years, includes the presence of plasmid quinolone resistance (PMQR) genes. The PMQR genes can be divided into three classes: the quinolone-specific effectors (QepA and OqxAB), the aminoglycoside acetyltransferase [AAC(6′)-Ib-cr], and the Pnr family. In *A. baumannii*, DNA gyrase and topoisomerase IV are shielded from ciprofloxacin inhibition by Qnr, which is encoded by *qnr*. Point mutations in the target location render bacteria with *qnr* prone to drug resistance (Higuchi et al., 2014). AAC(6′)-Ib-cr is a mutant of AAC(6′)-Ib, which acetylates free amino-containing quinolones.

### 2.4 Changes in outer membrane pore proteins

When their envelope’s permeability is altered, bacteria can become resistant to antibiotics, hampering their effectiveness frequently. Outer membrane protein (Omp) is a specialized channel protein located in the lipid bilayer structure either embedded in the cell membrane or on the cell membrane surface of gram-negative bacteria. Cell membrane permeability becomes altered when Omp is damaged, absent, or expressed at low levels. Consequently, drugs cannot bind to PBPs on the bacterial membrane, preventing them from inhibiting bacterial cell wall synthesis; this ultimately leads to drug resistance development.

Membrane pore proteins associated with *A. baumannii* drug resistance mainly include carbapenem-resistant associated out membrane protein (CarO), heat-modifiable protein (HMP), and OprD. In *A. baumannii*, resistance to β-lactamases is caused by high similarity among *A. baumannii* HMP, *P. aeruginosa* OmpF, and *Escherichia coli* OmpA. OXA23 carbapenemase interacts with OmpA and CarO, and this interaction may contribute to drug resistance (Wu et al., 2016). Resistance to amtronam, chloramphenicol, and nalidixic acid is also associated with OmpA (Smani et al., 2014). When *A. baumannii* generating OXA23 or OXA51 carbapenenase loses Omp29, its resistance to imipenem increases (Fonseca et al., 2013).

### 2.5 Alterations in PBPs

Bacterial cell walls are composed of PBPs. Carbapenems and other β-lactamases target D,D-transpeptidase (TP) in PBPs; this leads to the prevention of bacterial cell wall formation, followed by bacterial death (Cochrane and Lohans, 2020). In target bacteria, alterations in PBP structure or expression can lead to the loss of or reduction in β-lactam antibiotics’ binding affinity to PBPs; this makes the target bacteria resistant to β-lactam antibiotics. A decline in PBP2 expression is associated with bacterial carbapenem resistance. Toth et al. (2022) reported that PBP3 is necessary for *A. baumannii* survival, PBP1a inactivation results in partial cell lysis and bacterial growth retardation, and PBP2 inactivation amplifies these effects. The authors noted that the triple PBP1a/PBP1b/PBP2 mutant *A. baumannii* displayed a fourfold to eightfold increase in sensitivity to β-lactam antibiotics, whereas all other mutants demonstrated an increase in twofold to fourfold increase in sensitivity to β-lactam antibiotics. The simultaneous inhibition of PBP1a and PBP2 or the use of PBPs in combination with LdtJ, in addition to the critical PBP3, may be possible approaches for the development of novel therapeutics against *A. baumannii*.

### 2.6 Biofilm formation

A biofilm is a collection of bacteria and extracellular matrix, which adheres to the surfaces of biological tissues or nonbiological objects. It is a type of bacterial growth, similar to planktonic bacteria; it forms when bacteria secrete extracellular lipoproteins, polysaccharide matrix, fibrous proteins, and other substances wrapped around the bacteria themselves to create a microcolony structure. *A. baumannii* is a bacterium that often forms biofilms. The biofilm formation process is rapid, requiring approximately 24 h of cultivation *in vitro*, and involves five stages: reversible adhesion, irreversible attachment, microcolony formation, colonization or maturation, and dispersal (Pakharukova et al., 2018; Biswas and Mettlach, 2019). Biofilm resistance mechanisms include changes in permeability, inactivation of drugs via antimicrobial-inactivating enzymes immobilized on biofilm surface, nutrient and oxygen limitations, biofilm phenotype uniqueness, and immune evasion. The biofilm gene *bfmRS* has a hyperactive allele, which promotes resistance to serum complement killing, as well as tolerance to various antimicrobial medications via mechanisms including defense against attack by anti-β-amidase medicines (Geisinger et al., 2018). In extensively drug-resistant *A. baumannii*, drug resistance is strongly associated with its biofilm-associated genes *abaI*, *epsA*, *pglC*, *ompA*, and the class I integron gene. Moreover, biofilms may have the capacity to horizontally transfer genes, facilitating quick expression and migration of drug-resistant genes (Romero et al., 2008; Yang et al., 2019).

### 2.7 External discharge pump system overexpression

Efflux pumps constitute a unique efflux system found on bacterial outer membranes. The development of multidrug and extensive drug resistance development is facilitated by the presence of efflux pumps, which can remove pharmaceuticals from bacterial cells, lower antibiotic concentrations in bacterial cells, and diminish the medications’ bactericidal effects (Pagdepanichkit et al., 2016). Currently, five main types of efflux pump families are known to be associated with bacterial resistance: the ATP-binding cassette (ABC) superfamily, the small multidrug-resistant protein (SMR) family, the resistance nodulation division (RND) superfamily, the multidrug and toxic compound extrusion (MATE) superfamily, and the major facilitator (MFS) superfamily (Opazo et al., 2009; Coyne et al., 2011). Of these, the RND superfamily is the earliest discovered and most well-studied efflux pump family in *A. baumannii*. The RND superfamily, primarily consisting of AdeABC, AdeIJK, AdeDE, AdeXYZ, and AdeFGH efferent pump systems, can supply energy via the proton transmembrane concentration gradient (Nowak et al., 2015).

The AdeABC efflux pump system is the most crucial efflux system in *A. baumannii*. Some researchers have also suggested that *adeABC* is a marker for *A. baumannii* resistance. *adeABC*, encoding AdeA (membrane fusion protein), AdeB (intima efflux protein), and AdeC (outer membrane channel protein), is found in the chromosomal genome of *A. baumannii*. The AdeRS two-component regulatory system controls the AdeABC efflux pump system (Yoon et al., 2013). The upstream region of *adeABC* comprises *adeRS*, encoding the response regulator protein (AdeR) and the receptor protein (AdeS). AdeA, AdeB, and AdeC all demonstrate different expression levels. Although some studies have reported that AdeA has the highest detection rate among clinical isolates, PCR amplification data have revealed that AdeB has the highest detection rate (Modarresi et al., 2015). In general, AdeB is considered the most significant component of the AdeABC effusion pump system. Compared with *adeA*, *adeB* has a larger impact on carbapenem antibiotic resistance (Lee et al., 2010). The expression of the efflux pump gene *adeABC* is significantly higher in multidrug-resistant strains of *A. baumannii* than in drug-sensitive strains; moreover, *adeR* mutation alters the corresponding amino acids, indicating that the importance of *adeR* in multidrug resistance development (Chang et al., 2016). Lin et al. (2014) reported that *adeS* is the primary gene controlling the AdeABC system and that some point mutations in *adeS* can result in increased efflux pump production. In Germany, Meyer et al. (2022) reported that *A. baumannii* can resist the effects of disinfectants such as benzalkonium chloride, ethanol, and chlorhexidine *in vivo* by overexpressing the AdeABC efflux pump system.

Baumann–Acinetobacter RND superfamily proteins have been identified in two efflux pump systems, one of which is AdeIJK. The AdeIJK efflux pump system, a key mechanism underlying RND action in *Acinetobacter*, exhibits traits unique to each species. The AdeIJK efflux pump system can pump numerous antibiotics, including carbapenems, fluoroquinolones, tetracycline, lincomycin, neomycin, and tigecycline. All *Acinetobacter* spp. depend on AdeIJK to survive and maintain homeostasis; this is because AdeIJK plays essential roles in cells, such as regulating the lipid composition of cell membranes. AdeABC and AdeFGH have only been discovered in the Acinetobacter subgroup linked to infection (Darby et al., 2023). AdeJ becomes strongly expressed when *adeN*, the regulator gene upstream of *adeIJK*, becomes inactivated; this prevents antibacterials from entering bacteria.

AdeFGH, which can mediate the efflux of quinolones, macrolides, and tetracycline medicines but not aminoglycosides and β-lactamases, was initially described by French researchers in 2010. AlQumaizi et al. (2022) reported that AdeG may influence the sensitivity of *A. baumannii* to carbapenem. AdeFGH shares only 40% homology with AdeABC and AdeIJK. The 337 amino-acid–long region upstream of *adeFGH* is transcribed in a direction opposite to the direction in which AdeL, the transcription regulator of AdeFGH, is transcribed. Overexpression of the efflux pump gene *adeFGH* may be a result of changes in the *adeL* mutation locus (Coyne et al., 2010).

When AdeDE is overexpressed, the AdeDE efflux pump system—including the membrane fusion protein AdeD and the inner membrane efflux protein AdeE—can reduce the concentration of medications such as ceftazidime and rifampicin and thus increase drug resistance. Hou et al. (2012) reported the coexistence of AdeE and AdeB in a few *A. baumannii* isolates.

The MFS superfamily includes AmvA, CraA, TetA, and TetB. In *A. baumannii*, TetA mediates tetracycline resistance, whereas TetB mediates resistance to both tetracyclines and minocyclines (Lomovskaya et al., 2018). However, whether TetA and TetB induce drug resistance to tigecycline remains unknown. However, TetA and TetB do not cooccur in the same strain of *A. baumannii*. The TetA regulatory gene *tetR* encodes TetA repressor (Sumyk et al., 2021). In *A. baumannii*, a homolog of *E. coli* effector MdfA is linked to chloramphenicol resistance development in relation to CraA (Foong et al., 2019). Finally, in *A. baumannii*, disinfectant efflux is associated with the AmvA efflux pump (Rostami et al., 2021).

AbeM, a MATE family member, is an H^+^-linked multidrug efflux pump present in *A. baumannii*. The minimum inhibitory concentration (MIC) of drugs such as aminoglycosides, fluoroquinolones, sulfonamides, and chloramphenicol can be increased through the overexpression of the efflux pump gene *abeD* (Srinivasan et al., 2015). The SMR family member AbeS is clonally highly expressed in *E. coli*, and it is identical to EmrE in *E. coli*. AbeS can reduce cellular concentrations of neomycin, macrolides, mycotoxins, and fluoroquinolones in *E. coli* (Lytvynenko et al., 2016).

## 3 Mechanisms underlying tigecycline resistance

**Figure 2:**
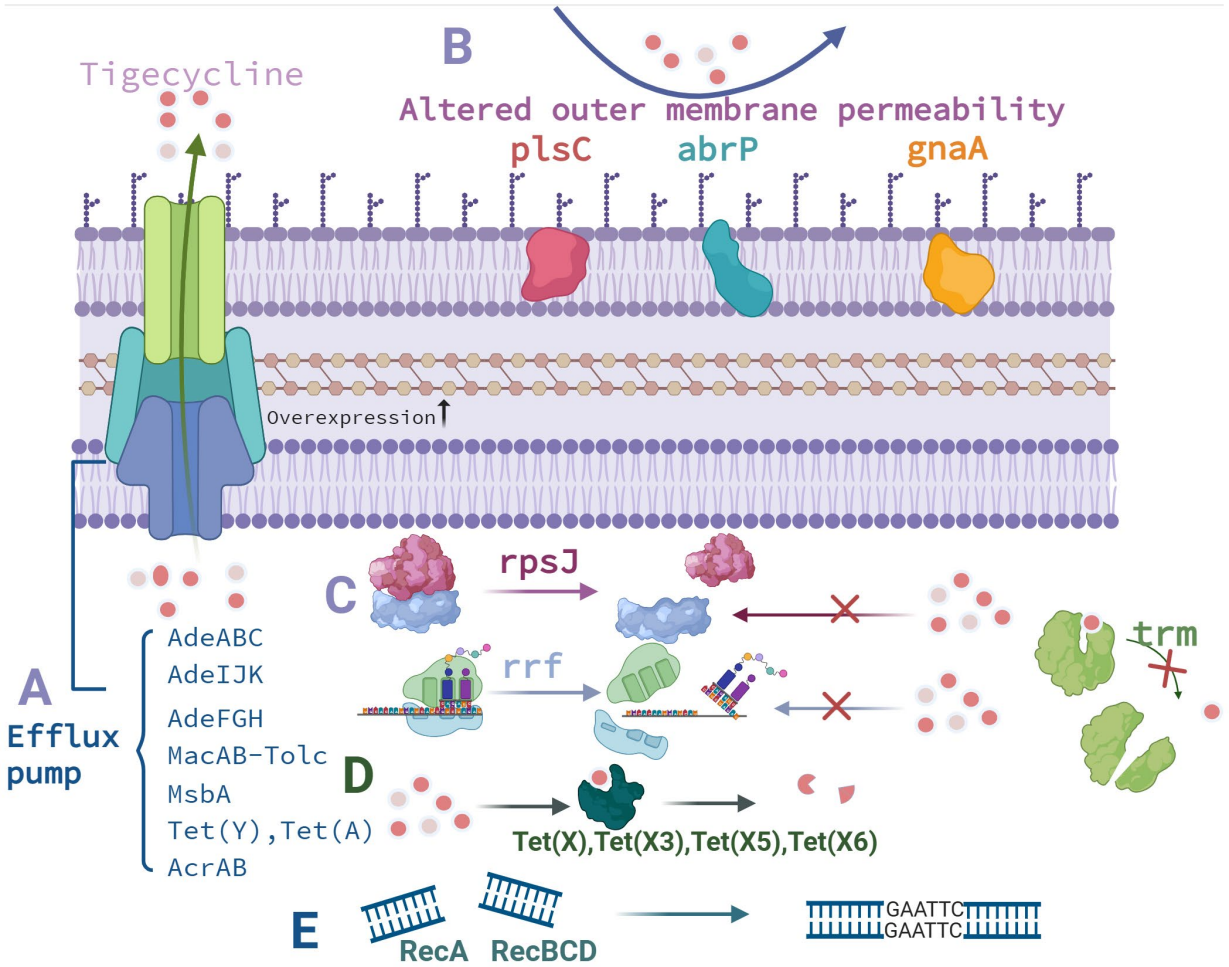
Mechanisms underlying tigecycline resistance in *A. baumannii*. **(A)** Influence of efflux pumps. **(B)** Outer membrane permeability alterations. **(C)** Drug target alterations. **(D)** Enzyme-mediated drug resistance modifications. **(E)** DNA damage–induced reactions. Created with BioRender.com.

Tigecycline, a third-generation tetracycline derivative, was the first glycylcycline antibiotic authorized for clinical use. Tigecycline—by attaching to the A position of 16S rRNA of the 30S subunit of the bacterial ribosome—mainly inhibits aminoacyl tRNA entry, peptide chain extension, bacterial protein synthesis, and bacterial growth. Tigecycline’s affinity for bacterial ribosomes is four times that of minocycline and more than 100 times that of tetracyclines. Therefore, tigecycline demonstrated a greater antibacterial activity and a wider antibacterial range—aiding it in overcoming TetM-mediated tetracycline resistance (Jenner et al., 2013). Although tigecycline demonstrates considerable antibacterial effects against *A. baumannii* despite the bacterium’s strong resistance potential and high viability, cases of tigecycline-resistant *A. baumannii* infections have been reported as early as 2007 (Peleg et al., 2007). Furthermore, the prevalence of *A. baumannii* resistance to tigecycline is increasing clinically. The emergence of the strains with direct or indirect resistance to tigecycline may have been due to the long-term selection pressure of various antibacterial drugs. The main mechanisms underlying tigecycline resistance in *A. baumannii* are classified into five categories: efflux pump overexpression, outer membrane permeability alterations, drug target alterations, modified enzyme–mediated resistance, and DNA damage induction (Table 1, Figure 2).

### 3.1 Influence of efflux pumps

The AdeABC efflux pump system was the first reported tigecycline resistance mechanism in *A. baumannii*. AdeABC overexpression results in reduced tigecycline susceptibility in *A. baumannii*. Currently, most studies agree that in *A. baumannii*, AdeB is crucial for the efflux of tigecycline via the AdeABC efflux pump. AdeB expression and the lowest inhibitory concentration of tigecycline are linearly associated in *A. baumannii* (Yuhan et al., 2016). Efflux pump inhibitors such as reserpine, PH-arg-β-naphthylamide dihydrochloride, and carbonyl cyanide 3-chlorophenylhydrazone (CCCP) have inhibitory effects and a tendency to increase tigecycline sensitivity by two to eight times in *A. baumannii* (Coyne et al., 2011).

During AdeABC exocycling, the two-component regulatory system AdeRS upstream of AdeABC plays a major role. In a South Korean study, the overexpression of AdeABC efflux pumps due to the ISAba1 insertion in *adeS* may have caused tigecycline resistance. However, the tigecycline-resistant subpopulation was unstable with continuous passage in antibiotic-free conditions. The addition of ISAba10 to AdeR and nucleotide modification of AdeS in some mutants may be responsible for the reversal of tigecycline sensitivity via an antibiotic-free passage (Jo and Ko, 2021). Some AdeS mutations, such as G186V and A1a94val, are associated with AdeABC overexpression (Hornsey et al., 2010; Salehi et al., 2021). The strong expression of AdeABC is regulated by another two-component regulatory system called BaeSR (Lin et al., 2014). However, additional relevant research determining the relationship between BaeSR and AdeRS is needed.

All strains of *A. baumannii* possess the AdeIJK efflux pump system, which may be involved in the bacterium’s endogenous resistance process. In *A. baumannii*, when AdeIJK is inhibited, the MIC of tigecycline decreases by three to eight times. Similarly, when AdeABC is inhibited, tigecycline’s MIC decreases by 85 times. These observations indicate that AdeABC and AdeIJK function concurrently to reduce tigecycline resistance. Notably, growth inhibition increases with an increase in the amount of inducer in *A. baumannii* when the *adeIJK* operon is cloned and overexpressed. In particular, *adeIJK* overexpression leads to growth suppression and deleterious reactions in *A. baumannii* (Damier-Piolle et al., 2008). The AdeIJK regulator gene, *adeN*, has been noted to have three amino acid mutations in a whole genome sequence of tigecycline-resistant *A. baumannii*; moreover, one-third of *A. baumannii* isolates have been noted to demonstrate an ISAba1 insertion, resulting in the aforementioned *adeN* mutations. Furthermore, *adeJ* expression is upregulated by two to six times (Gerson et al., 2018). In general, AdeN may inhibit the *adeIJK* operon in tigecycline-sensitive *A. baumannii*.

AdeL is the transcription factor regulating the AdeFGH efflux pump upstream, and point mutations in *adeL* can increase *adeG* expression up to 100-fold (Coyne et al., 2010). However, Deng et al. (2014) noted that drug-resistant and -sensitive *A. baumannii* demonstrate similar levels of *adeG* expression. Certain strains exhibiting elevated *adeFGH* expression do not exhibit an *adeL* mutation. Therefore, transcription factors other than AdeL may regulate *adeFGH* expression in *A. baumannii*. Moreover, *adeFGH* expression is negatively regulated by SoxR in *A. baumannii*; therefore, *soxR* overexpression may lead to increased tigecycline sensitivity in the bacterium (Li et al., 2017).

In contrast to other efflux pump systems, ABC uses energy through ATP-bound hydrolysis to perform pumping tasks. Hammerstrom et al. (2015) noted that MsbA in a tigecycline-resistant *A. baumannii* group was substantially different from that in other groups; the authors concluded that the differences were due to functional changes that facilitated tigecycline efflux. However, Chen et al. (2014) did not observe any discernible variations in the effects of MsbA on tigecycline resistance. Studies confirming MsbA’s effects on tigecycline are therefore required. Tigecycline-resistant *A. baumannii* has been noted to have considerably elevated expression of MacB, which is part of the MacAB-TolC efflux pump system (Lin et al., 2017a). Further research determining the effects of the MacAB-TolC efflux pump system on tigecycline resistance, as well as the underlying mechanisms, in *A. baumannii* is warranted. Wang et al. (2021) discovered *tet(Y)*, the first tigecycline resistance-related gene associated with plasmids, in China. The introduction of plasmid p2016GDAB1 containing *tet(Y)* through electroporation resulted in a 16-fold increase in the MIC of tigecycline, whereas *tet(Y)* overexpression on a 72,156-base pair (bp) plasmid increased it by twofold to fourfold. Furthermore, the authors noted that p2016GDAB1 contained the regulatory gene *tetR*, which increased the MIC of tigecycline by 128 times after regulating *tet(Y)* and *tetA(39)* transcription. Strong tigecycline resistance may be a result of combined mediation by Tet(Y) and TetA(39). Liu et al. (2023) indicated that their screened tigecycline-resistant *A. baumannii* 17978R became resistant to tigecycline, possibly after the loss of plasmids containing *hns* or after the insertion of *hns* through an insertion sequence. This possibly increased TonB expression, which then increased the efflux pump expression. Furthermore, insertion sequence insertion may have resulted in the deletion of *acrR*, encoding AcrR, which inhibits the efflux pump AcrAB and thus increases efflux pump expression.

### 3.2 Modification of enzyme-mediated drug resistance

Tet(X) is a riboflavin-dependent monooxygenase, initially identified in *Bacteroides tenuis* transposons Tn4351 and Tn4400. NADPH and Mg^2+^ cause tigecycline hydroxylation and inactivation. The Tet(X) family, encoded by eight genes, is associated with considerable levels of tigecycline resistance. Tet(X), Tet(X3), Tet (X5), and Tet(X6) can increase tigecycline resistance in *A. baumannii* (Cui et al., 2021). Tet(X3) renders all tetracyclines—including tigecycline, as well as the recently FDA-approved elacycline and omacycline—are inactive; this increases the MIC of tigecycline by 64–128 times. Through hydroxylation of Tet(X5) and Tet(X6), resistance to many antimicrobials, including tigecycline, can be induced in *A. baumannii*. Finally, translocation and recombination mediated by IScr2, a recently discovered insertion sequence, IScr2, accelerate the spread of Tet(X5) (He et al., 2019; Chen et al., 2021).

### 3.3 Outer membrane permeability alterations

1-Acyl3-glycerol phosphate acyltransferase, encoded by *plsC*, is involved in bacterial outer membrane formation. Li et al. (2015) induced drug resistance in clinical isolates of tigecycline-sensitive *A. baumannii*, yielding a drug-resistant strain called 19,606-M24, and noted that *omp38*, *hp*, and *plsC* are the possible resistance genes based on their whole genome sequencing results. In their follow-up studies, the authors concluded that only frameshift mutation in *plsC* is linked to tigecycline resistance and, through flow cytometry, that the *plsC* mutation leads to changes in the strain’s cell membrane. Li et al. (2016) also discovered *abrP*, a gene linked to resistance to drug, including tigecycline. The mutant’s phenotype could be recovered by introducing wildtype *abrP*. The authors also discovered that the *abrP*-deficient mutant had enhanced cell membrane permeability, which reduced its cell growth rate. Taken together, these results confirmed that *abrP* is crucial for *A. baumannii* resistance and adaptation.

Tigecycline resistance is also associated with *gnaA*. In particular, Xu et al. (2019) discovered *gnaA* inserted by ISAba16 transposons in MDR-ZJ06M, a multidrug-resistant and virulent *A. baumannii* strain. The isolates had significantly increased tigecycline resistance. However, the supplementary experiment revealed no change in MICs of tigecycline. Therefore, further experimental evidence demonstrating the role of *gnaA* in tigecycline resistance development is required.

### 3.4 Drug target alterations

*rpsJ* encodes the ribosome S10 protein. Mutation in *rpsJ* causes the S10 protein ring to shift structurally, in turn altering the shape of 16S rRNA and reducing the affinity between tigecycline and ribosome (Beabout et al., 2015). *trm* encodes S-adenosyl-L-methionine-dependent methyltransferase (AdoMet). Through whole genome sequencing, Chen et al. (2014) identified mutant *trm* in the *A. baumannii* strain 19606-T8 (MIC = 8 mg/L), experimentally engineered to develop tigecycline resistance. Although AdoMet has been hypothesized to be crucial for moderate tigecycline resistance induction, how it mediates the loss of sensitivity to tigecycline remains unknown. In *A. baumannii*, tigecycline resistance is strongly influenced by mutations in *trm*, and tigecycline-resistant strains with *trm* mutations exhibit various amino acid alterations, including M378K, K291R, H312P, and S94A (Ghalavand et al., 2022). However, in both tigecycline-resistant and -sensitive *A. baumannii*, Zheng et al. (2023) noted mutations in *trm* and *plsC* but not in *rpsJ*. As such, additional studies confirming the influence of mutations in *trm*, *plsC*, and *rpsJ* on the emergence of tigecycline resistance in *A. baumannii* are warranted.

*rrf* encodes ribosome recycling factor (RRF). Hammerstrom et al. (2015) discovered a mutation in *rrf* in tigecycline-resistant *A. baumannii*; the authors hypothesized that this mutation reduces mRNA translation, affects tigecycline and ribosome binding, and eventually, results in tigecycline resistance. Hua et al. (2021) confirmed this hypothesis through Western blotting and multiribosome spectrum analysis: mutations in *rrf* reduced RRF expression, affecting the ribosome cycle process. Through an *in situ* complementarity experiment, the authors also demonstrated that mutations in *rrf* were associated with tigecycline resistance in *A. baumannii*.

### 3.5 DNA damage–induced reactions

Antimicrobials can cause bacterial death via DNA damage. However, RecA and RecBCD together can repair this damage. RecBCD is crucial for DNA double-stranded break repair, whereas RecA is the primary enzyme involved in homologous recombination and recombination repair. RecA also contributes to stress and virulence responses in *A. baumannii*. Ajiboye et al. (2018) performed deletion experiments with recA and recBCD in *A. baumannii* and noted considerable reductions in the bacterium’s tigecycline, gentamicin, and polymyxin MICs. These results indicated that the recA- and recBCD-mediated repair pathways may protect *A. baumannii* from the effects of antimicrobials, eventually resulting in the development of drug resistance.

## 4 Mechanisms underlying polymyxin resistance

*Bacillus polymyxa* produces polymyxins, which are polypeptides with antibacterial properties and can be divided into classes A, B, C, D, and E. Polymyxin B and colistin are the most frequently prescribed medications in clinical practice (Cassir et al., 2014). In the 1950s, polymyxins were used for the first time in clinical settings. Because their low toxicity and comparable bactericidal and antibacterial spectra, polymyxins B and E are frequently used. Some multidrug-resistant gram-negative organisms, such as *K. pneumoniae* and *P. aeruginosa*, can cause pulmonary infections (Lenhard et al., 2019). However, the use of polymyxins has been since restricted because they may cause neurotoxicity and nephrotoxicity.

The recent rise of multidrug-resistant, extensively drug-resistant, and pandrug-resistant bacteria has led to a renaissance of antimicrobials for the discovery of drugs of last resort. Moreover, additional clinical studies have focused their attention on polymyxin use (Carrasco et al., 2021). Polymyxins primarily function as a bactericide by disrupting the lipopolysaccharide (LPS) structure in the outer membranes of gram-negative bacteria. Negatively charged lipid A in LPS interacts with positively charged polymyxin residues such as 4-amino-L-arabinose (l-Ara4N) and phosphoethanolamine (PEtN). Colistin can bind with these negatively charged phosphate groups in place of Ca^2+^ and Mg^2+^. Because LPS has a higher affinity than bivalent cations, its stability is compromised. This leads to the destruction of the bacterial outer membrane, a decrease in the serous membrane’s surface tension, an increase in its permeability, the loss of the serous membrane’s barrier function, and the leakage of bacterial contents, eventually resulting in bacterial death (Jeannot et al., 2017).

For multidrug-resistant *A. baumannii*, polymyxin use is one of the few remaining treatments; in particular, it is used as a drug of last resort for life-threatening infections. Polymyxin-resistant *A. baumannii* has emerged with the increase in the clinical use of polymyxins. A recent meta-analysis revealed that the polymyxin resistance rate of *A. baumannii* was higher in Southeast Asian and eastern Mediterranean countries than in other countries (totaling 11.2%); it was the highest in Lebanon (17.5%) and China (11.8%) but the lowest in Germany (0.2%) (Pormohammad et al., 2020). The prevalence of polymyxin-resistant strains has increased due to increased polymyxin use as a rescue treatment for multidrug-resistant *A. baumannii* infections. However, a comprehensive set of standardized practices or conventional treatment recommendations for polymyxin use is unavailable in different countries or regions. In an epidemiological investigation of polymyxin-resistant *A. baumannii*, the multilocus sequence typing subtypes ST92 and ST208 were noted to be identical. Therefore, polymyxin-resistant *A. baumannii* strains may have spread significantly throughout Europe, Asia, and North America (Novović and Jovčić, 2023).

**Figure 3:**
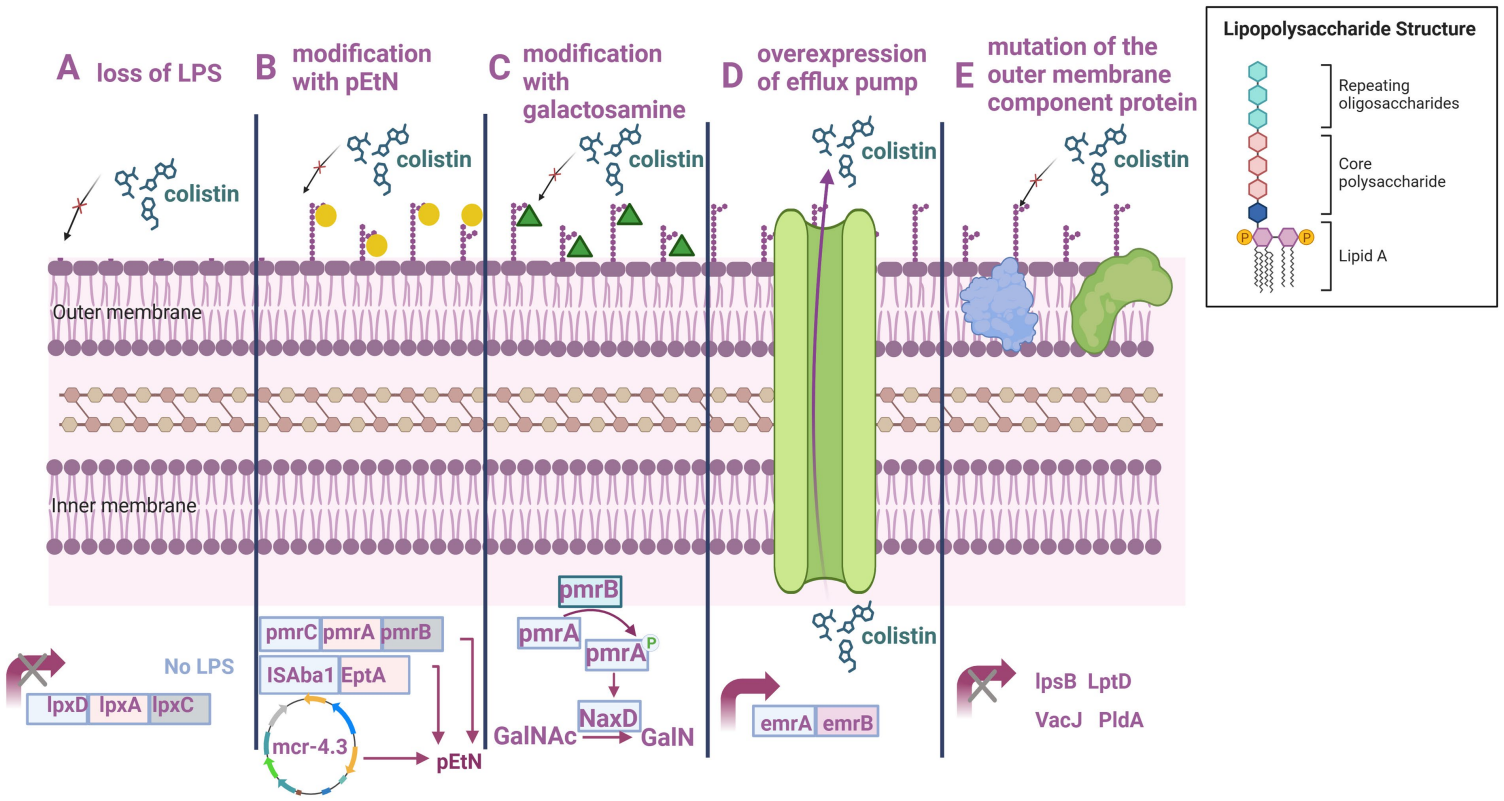
Mechanism underlying polymyxin resistance in *A. baumannii*. **(A)** LPS loss. **(B)** PEtN modification. **(C)** Galactosamine modification. **(D)** Efflux pump overexpression. **(E)** Outer membrane component protein mutations. Created with BioRender.com.

The mechanisms underlying polymyxin resistance in *A. baumannii* are categorized as follows: LPS and lipid A loss, LPS structure modification, plasmid-mediated polymyxin resistance, efflux pump resistance mechanism, and outer membrane component protein mutations (Table 1, Figure 3).

### 4.1 LPS loss

Gram-negative bacteria have long been thought to require LPS production to survive (Zhang et al., 2013). Only a few bacteria, including *A. baumannii*, *Moraxella kataxa*, and *Neisseria meningitida* (Steeghs et al., 1998; Peng et al., 2005; Moffatt et al., 2010), have been found to thrive without LPS thus far. Mutation in *lpxA*, *lpxC*, or *lpxD*—involved in lipid A production—leads to the complete disappearance of LPS in *A. baumannii*. Through the addition of an insertion sequence element from the IS4 family (which is comparable to the ISX03 element), Moffatt et al. (2011) discovered that *lpxD* is deleted in resistant strains of *A. baumannii*, which resulted in LPS loss and resistance development. Moreover, the authors discovered that ISAba11 inactivated *lpxA* and *lpxC* of *in vitro* polymyxin resistance variants in *A. baumannii* ATCC19606. Lipid A biosynthesis is caused by the deletion, substitution, and insertion of different *lpxA*, *lpxC*, and *lpxD* nucleotides, and the combination of mutations in *lpxC*, *lpxD*, and the *pmrAB* operon may result in a synergistic effect of polymyxin resistance. However, some drug-resistant bacteria demonstrated *lpxA*, *lpxC*, and *lpxD* downregulation, reducing LPS production (Thi Khanh Nhu et al., 2016; Nurtop et al., 2019; Jovcic et al., 2021). *In vitro*, the LPS-deficient mutant strain developed slower and was less pathogenic than the wildtype strain (Wand et al., 2015; Carretero-Ledesma et al., 2018).

*lpx* mutant strains are more vulnerable to lysozyme released by neutrophils and are more susceptible to neutrophil cell-mediated killing. LPS loss may expand the pathways through which lysozyme can enter periplasmic peptidoglycans, facilitating lysozyme targeting and increasing antimicrobial activity against LPS-deficient collisin-resistant bacterial strains (Kamoshida et al., 2020). LPS-deficient *A. baumannii* demonstrates greater vulnerability to other antimicrobials such as carbapenems, quinolones, tigecycline, and rifampicin despite appearing to have strong polymyxin resistance. It also demonstrated greater susceptibility to certain disinfectants, including ethanol, chlorhexidine, and phenol-based cleaners.

Moderate fitness loss is linked to the absence of LPS. LPS loss reduces *A. baumannii*’s pathogenicity and fitness and leads to variations in biofilm development and viability under iron restriction. LPS-deficient *A. baumannii* demonstrates alterations in signaling through toll-like receptors and enhances susceptibility to host antimicrobial peptides, suggesting that host factors contribute to decreased fitness (Moffatt et al., 2013). Reduced biofilm development after LPS deletion increases the possibility of the affected strains lacking the necessary biofilm-forming mechanism component. The biochemical mechanism underlying the considerable abnormalities resulting from LPS deficiency under iron restriction. A mechanism underlying this outcome may involve LPS deficiency leading to changes in the membrane composition of *A. baumannii*, limiting its ability to import extracellular iron (Carretero-Ledesma et al., 2018; Kamoshida et al., 2020, 2022).

### 4.2 LPS structure modifications

The addition of PEtN to lipid A in *A. baumannii* is crucial for polymyxin resistance development. Phosphate groups added to PEtN at both the 4′ and 1′ locations cause the LPS component to have a negative charge and reduce PEtN’s propensity for binding polymyxin (Olaitan et al., 2014). Alterations in the two-component regulatory system PmrAB causes overexpression of the PEtN transferase PmrC, resulting in polymyxin resistance (Potron et al., 2019). Polymyxin-sensitive strains can alter into polymyxin-resistant phenotypes as a result of partial deletion mutations in PmrB. PmrCAB expression may be increased as a result of a mutation in PmrB through PmrA activation (Adams et al., 2009). Mutations in PmrA alone also increase *A. baumannii*’s resistance to polymyxin (Sun et al., 2020). However, in some instances, little to no link has been noted between PmrAB and high PmrC expression (Kabic et al., 2023). Therefore, PmrC may be regulated by regulatory mechanisms other than those of PmrAB. Few studies have examined the connection between mutations in PmrC and polymyxin resistance thus far. I42V and L150F, two prevalent *pmrC* variants, may be linked to polymyxin resistance (Nurtop et al., 2019). *A. baumannii* is more resistant to polymyxin when PmrC R125P mutations and PmrB alterations are combined (Gerson et al., 2020). Gerson et al. (2019) revealed that three polymyxin resistance-related mutations in PmrB cause PmrC expression to increase or decrease, demonstrating that PmrB is not the only protein regulating *pmrC* expression in *A. baumannii*.

Clinical *A. baumannii* strains exhibit significant levels of polymyxin resistance as a result of ISAba125 insertion into the gene encoding the global regulator H-NS. The expression of the distal gene is triggered by the insertion of the insertion sequence element in this gene, which is another PmrC homolog called phosphoethanolamine transferase (EptA), the product of which shares 93% homology with PmrC. Although *eptA* alone in the bacterial genome cannot impart resistance to polymyxin, the integration of ISAba1 upstream of *eptA* might result in an increase in the enzyme’s production (Trebosc et al., 2019).

In a Chinese study, ISAba1 insertion was noted to occur between 13 and 18 bp upstream of *eptA*. The authors hypothesized that *eptA* expression is not dependent on the distance between ISAba1 and *eptA* transcription initiation. In *A. baumannii*, EptA is activated more directly when ISAba1 is inserted upstream of *eptA*, leading to polymyxin resistance. Moreover, under the strain of continuous polymyxin therapy, random insertion events may lead to an increase in the frequency of polymyxin resistance in the future (Ye et al., 2023). Despite the absence of L-Ara4N, the sensor kinase PmrB regulates NaxD deacetylase, a major mediator of lipid A alteration resulting in polymyxin resistance, in *A. baumannii* (Chin et al., 2015).

### 4.3 Plasmid-mediated polymyxin resistance

The plasmid-mediated gene *mcr*, encoding a PEtN transferase, was first discovered in a Chinese strain of polymyxin-resistant *E. coli* SHP45, isolated from a pig with *Mcr1*. In *E. coli*, Mcr added phosphoethanolamine to lipid A, producing mutagenic polymyxin resistance (Liu et al., 2016). Thus far, 10 *mcr* genes, ranging from *mcr-1* to *mcr-10*, have been reported in the environment, animals, food, and humans (Hussein et al., 2021). In Brazil, a meningitis patient’s cerebrospinal fluid was noted to include *A. baumannii* contained with *mcr-4.3* in 2008. This gene can maintain polymyxin-resistant resistance through LPS modification. The pET-26b-*mcr-4.3*-transformed *E. coli* was noted to develop polymyxin resistance (Martins-Sorenson et al., 2020). However, in South Africa, recombinant expression of *mcr-4.3* in *A. baumannii* did not confer polymyxin resistance in *E. coli* (Snyman et al., 2021). pAB18PR065, harboring *mcr-4.3*, could not be transferred in the splicing, transformation, and electroporation assays (Ma et al., 2019). An *mcr-4.3*-carrying *A. baumannii* strain has also been isolated from pig feces; however, further related research is required. *A. baumannii* harboring *mcr-1*, *mcr-2*, and *mcr-3* was also discovered in Iraq (Al-Kadmy et al., 2020).

### 4.4 Drug resistance due to efflux pumps

Laboratory-induced polymyxin-resistant *A. baumannii* demonstrated a 1.6 times increase in expression of EmrB from the EmrAB efflux pump system. *A. baumannii* becomes more sensitive to polymyxin after *emrB* deletion. These results indicate that the EmrAB efflux pump system reduces *A. baumannii*’s susceptibility to polymyxin. EmrAB efflux pump system has been postulated to be a crucial mechanism underlying polymyxin resistance through pumping and cytoplasmic acidification; this result requires further confirmation. The aforementioned mechanisms are similar to those of the efflux pump/potassium antiporter system, which mediates polymyxin resistance in *Yersinia* when induced at higher temperatures (≥37°C) (Lin et al., 2017a). Wiradiputra et al. (2023) reported that *A. baumannii* with low polymyxin resistance demonstrates at least two times increase in *emrB* and *ompW* expression. Ni et al. (2016) noted that using the efflux pump inhibitor CCCP considerably reduces the MIC of polymyxin in *A. baumannii*. These results further confirm that efflux pump systems contribute to polymyxin resistance.

### 4.5 Outer membrane component protein mutations

Mutations in some non-LPX proteins that make up the outer membrane of *A. baumannii* also contribute to increased polymyxin resistance. *lpsB* encodes a glycosyltransferase, involved in LPS synthesis. Mutations in *lpsB* lead to a reduction in polymyxin permeability (Hood et al., 2013). Mutations in *lpsB* identified thus far include H181Y, 241 K, D146G, G218H, E219S, and T331I (Lean et al., 2014; Dafopoulou et al., 2015; Yang W. et al., 2023; Yang Y. et al., 2023). Mutations in *lptD* also interfere with LPS translocation to the outer membrane, resulting in moderate polymyxin resistance in *A. baumannii* (Bojkovic et al., 2015). A Vietnamese study concluded that mutations in the lipoprotein VacJ and the phospholipase PldA—believed to maintain the lipid asymmetry of the outer membrane—lead to polymyxin resistance in *A. baumannii* (Thi Khanh Nhu et al., 2016). Lipid metabolism is a major pathway. Polymyxin acts by disrupting the membrane, and *lpsB* mutant strains are defective in the production of main outer membrane components; therefore, the effects of polymyxin and *lpsB* mutation on lipid metabolism overlap significantly (Hood et al., 2013).

## 5 Conclusions and future prospects

Nonstandardized use of antibiotics has led to the development of antibiotic resistance among pathogenic microorganisms. By 2050, diseases due to antibiotic-resistant bacteria may lead to 10 million deaths annually (Carr et al., 2019). In most hospitals worldwide, *A. baumannii* causes nosocomial infections, including bloodstream infections, wound infections, urinary tract infections, meningitis, and pneumonia associated with ventilator use. *A. baumannii* bloodstream infections are associated with a high mortality rate. Drug-resistant *A. baumannii* can possess nearly all bacterial resistance pathways along with a considerably unlimited capacity for antibiotic resistance development. *A. baumannii* can produce all forms of β-lactamases. Moreover, carbapenem-resistant *A. baumannii* (CRAB) detection rate remains high. Almost all *A. baumannii* strains discovered thus far express aminoglycoside-altering enzymes. Numerous clinical isolates have exhibited a high level of different efflux pumps. The related resistance genes have been noted to inhibit the bactericidal activity of aminoglycosides, carbapenems, quinolones, and broad-spectrum cephalosporins. Because of their comparatively low rates of resistance, tigecycline and polymyxin are considered drugs of last resort for *A. baumannii* infection treatment. However, with the annual increase in tigecycline and polymyxin use, the number of cases of infection due to *A. baumannii* resistant to these antibiotics is increasing globally. Furthermore, polymyxins have some nephrotoxicity and neurotoxicity, and tigecycline can result in notably low plasma concentrations when following the tigecycline instructions recommended dosing regimen. In general, given the availability of additional efficient treatment options, tigecycline and polymyxin should not be administered alone.

Because of the dearth of solid clinical evidence justifying the use of any one medication therapy, antibiotics are frequently used in combination for the treatment of illnesses with high mortality rates. Medicines with antibacterial activity at first may cause resistance, and patients with a CRAB infection are typically extremely sick with more consequences. Therefore, two or more active medications should be used in all applicable situations (Tamma et al., 2022). Combination regimens based on polymyxin, tigecycline, and sulbactam have often been reported. For instance, sulbactam-based combinations (polymyxin + sulbactam or tigecycline + sulbactam) had better synergistic effects than polymyxin + tigecycline (Qu et al., 2020). However, no combination regimen can reduce mortality significantly or improve clinical response considerably. Therefore, more clinical and *in vivo* study data are needed to explore effective combination regimens.

Few novel medications specifically targeting CRAB are currently available. For instance, cefiderocol, a novel ferriferous cephalosporin that penetrates the cell membrane of gram-negative bacteria via a unique route, is highly effective at eliminating all gram-negative bacteria. To reach the cytoplasm at a large concentration, cefiderocol forms complexes with trivalent iron ions and, via the bacterial ferritransporter, becomes transported through the outer cell membrane into the inner wall of the cell. There, by binding to the receptor, cefiderocol prevents cell wall synthesis (Domingues et al., 2023). Eravacycline, a synthetic tetracycline that, similar to other tetracyclines, binds to the 30S ribosome subunit of bacteria and suppresses protein synthesis. Similar to that of tigecycline, eravacycline’s activity is not influenced by ribosome-protective proteins (Hetzler et al., 2022). Durlobactam, a novel diazadicycloctanone β-lactamase inhibitor, effectively inhibits class A, C, and D β-lactamases (Papp-Wallace et al., 2023). Dulobactam + sulbactam have been noted to effectively restore the sulbactam sensitivity of CRAB clinical isolates (August et al., 2023). New-generation polymyxin-type antibacterial adjuvants, including SPR206, MRX-8, and QPX9003, are also being assessed in the clinical trial stage (Aslan et al., 2022). However, certain drug-resistant *A. baumannii* have been discovered during the development of new drugs, including cefiderocol. In other words, cautious prescription and consistently monitored use of these novel antibiotics, along with regular assessment of bacterial drug resistance, are highly warranted.

Since the beginning of the postantibiotic era, the focus of contemporary research on novel therapeutics for *A. baumannii* infections has moved from antibiotic production to that of nonantibiotic compounds. Medications targeting virulence factors can reduce pathogenicity or improve sensitivity by inhibiting *A. baumannii* or its virulence factors. Medications that do not produce an excessive load on bacterial metabolism are less likely to induce resistance in the target bacteria (Saipriya et al., 2020). Therapies for *A. baumannii* infections currently under development include immunotherapeutics (Buchhorn de Freitas and Hartwig, 2022), photodynamic therapy (Bustamante and Palavecino, 2023), subunit vaccine therapy (Yang et al., 2022), phage therapy (Tan et al., 2022), and antimicrobial peptide therapy (Rangel et al., 2023). Research in other fields may also indirectly influence future *A. baumannii* treatments.

## Author contributions

JS: Visualization, Writing – original draft, Writing – review & editing. JC: Visualization, Writing – review & editing. SL: Writing – review & editing. YZ: Writing – review & editing. MZ: Writing – review & editing.
